# Evaluation of a quality control phantom for digital chest radiography

**DOI:** 10.1120/jacmp.v2i2.2621

**Published:** 2001-03-01

**Authors:** Eugene Mah, Ehsan Samei, Donald J. Peck

**Affiliations:** ^1^ Department of Radiology Medical University of South Carolina Charleston South Carolina 29425; ^2^ Department of Radiology Duke University Medical Center Durham North Carolina 27710; ^3^ Department of Radiology Henry Ford Hospital Detroit Michigan 48202

**Keywords:** digital radiography, quality control, chest phantom

## Abstract

*Rationale and Objectives*: To examine the effectiveness and suitability of a quality control (QC) phantom for a routine QC program in digital radiography. *Materials and Methods*: The chest phantom consists of copper and aluminum cutouts arranged to resemble the appearance of a chest. Performance of the digital radiography (DR) system is evaluated using high and low contrast resolution objects placed in the “heart,” “lung,” and “subdiaphragm” areas of the phantom. In addition, the signal levels from these areas were compared to similar areas from clinical chest radiographs. *Results*: The test objects included within the phantom were effective in assessing image quality except within the subdiaphragm area, where most of the low contrast disks were visible. Spatial resolution for the DR systems evaluated with the phantom ranged from 2.6 lp/mm to 4 lp/mm, falling within the middle of the line pair range provided. The signal levels of the heart and diaphragm regions relative to the lung region of the phantom were significantly higher than in clinical chest radiographs (0.67 versus 0.21 and 0.28 versus 0.10 for the heart and diaphragm regions, respectively). The heart‐to‐diaphragm signal level ratio, however, was comparable to those in clinical radiographs. *Conclusion*: The findings suggest that the attenuation characteristics of the phantom are somewhat different from actual chests, but this did not appear to affect the post‐processing used by the imaging systems and usefulness for QC of these systems. The qualitative and quantitative measurements on the phantom for different systems were similar, suggesting that a single phantom can be used to evaluate system performance in a routine QC program for a wide range of digital radiography systems. This makes the implementation of a uniform QC program easier for institutions with a mixture of different digital radiography systems.

PACS number(s): 87.57.–s, 87.62.+n

## I. INTRODUCTION

Utilization of digital radiography (DR) in radiology departments is becoming increasingly widespread. Benefits of digital radiography include reduced costs associated with film developing and handling, increased dynamic range of the acquired image, and reduced repeat rate. Digital storage of the acquired images also provides the ability to perform image manipulation and long‐term image archiving. Images can be made widely available to remote locations for display or diagnosis over computer networks. Realizing and maintaining these benefits requires the implementation of an effective quality control (QC) program. Quality control for digital radiography should be considered as essential as a quality control program for film processors. A QC program should include routine testing and inspection of the digital radiography components [e.g., imaging detectors), cassettes, plate readers, etc.] performed daily, weekly, and annually.^1^ Control limits on various imaging parameters related to image quality (e.g., exposure indicator, signal‐to‐noise ratio, and spatial resolution) also need to be established.^2^ The results of the QC tests should be documented and evaluated for any trends occurring overtime.

In order to be practical, the tests in a QC program should be relatively easy to perform and not require detailed or complicated setup procedures. To accomplish this, a chest phantom has been developed^3^ with embedded test objects to evaluate the resolution and contrast detectability of digital radiography systems. This study involves the evaluation of the phantom and its characteristics under different imaging conditions with six different digital radiography systems in order to assess the usability of the phantom as part of a routine QC program.

## II. MATERIALS AND METHOD

### A. Phantom construction

**Figure 1:**
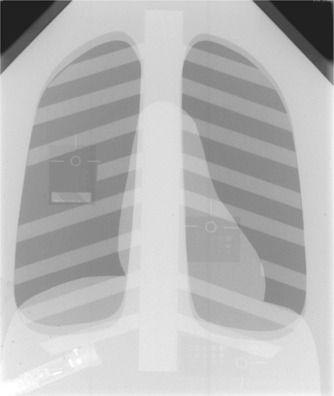
Radiographic image of the Nuclear Associates digital chest radiography phantom. Test objects embedded in the phantom are visible in the lung, heart, and subdiaphragm area. The Radcal 10×5–6 ionization chamber is visible in the lower left corner of the image.

A phantom for digital chest radiography^3^ (Nuclear Associates Model 07‐646, Nuclear Associates, Carle Place, NY) was assessed for performance and ability to perform routine evaluation of image quality on digital imaging systems. The chest phantom was pseudoanthropomorphic in that it was designed to resemble the appearance of chest radiographs while including various objects for assessing image quality. This allowed the image processing software to treat the resulting image as a chest image and thus to facilitate producing reproducible results and mimic the clinical utilization of the digital system (Fig. 1).

The phantom was constructed from layers of 0.5‐mm thick copper and 6‐mm thick aluminum sheets cut into shapes resembling the heart, diaphragm, spine, and ribs, and a copper sheet with cutouts in the shape of the lung fields. The components were arranged to produce an image that resembled a chest radiograph. A wire mesh covering the phantom served to broaden any peaks in the image histogram resulting from large areas of uniform exposure. The entire phantom was sandwiched between 2.5‐cm thick acrylic sheets to provide additional attenuation.

**Figure 2:**
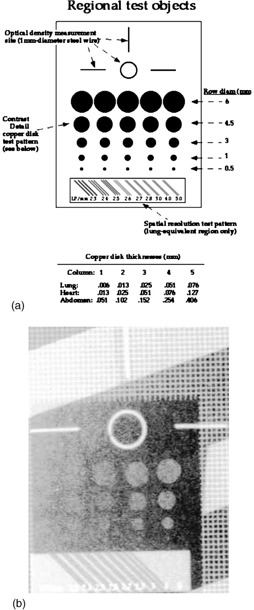
(a) Schematic of the image quality test objects found in the phantom^3^ (reproduced with permission). (b) Radiograph of the quality assessment test objects.

Within the phantom were three test objects for performing a subset of tests recommended by AAPM Task Group #10.^1^ The line pair test object was located in the lung region and consisted of nine line pair groups ranging from 2.3 to 5 lp/mm oriented at 45° [Fig. 2(a)]. The purpose of this test object is to evaluate the effect of changes to the laser, optics, or scanning subsystems on spatial resolution. For a properly operating and calibrated digital radiography system, the range of line pairs that are provided by the test object should be capable of measuring the Nyquist frequency for most currently available systems. Although the line pair pattern is limited to measuring the limiting resolution of the DR system (ideally this is the Nyquist frequency), degradation in the operation of the laser, optics, or scanning subsystems significant enough to visibly affect image quality should be reflected by changes in the number of line pairs visible. A comprehensive evaluation of the resolution properties of a digital radiography system would require a measurement of the system MTF, which would be beyond the scope of a routine QC program.

Objects to evaluate contrast detail sensitivity and signal level [Figs. 2(a) and 2(b)] were located in the lung, heart, and subdiaphragm region of the phantom. The contrast sensitivity objects were composed of copper disks of varying thickness and size. Copper disk thickness ranged from 0.006 to 0.076 mm in the lung, 0.013 to 0.127 mm in the heart, and 0.051 to 0.406 mm in the subdiaphragm area. Disk diameters ranged from 0.5 to 6 mm. This provided a range of contrast detail combinations specific to each region for assessing the contrast detail sensitivity of the digital imaging system. A loop of wire in each test object provided a reference region of interest (ROI) area to obtain optical density or mean pixel value measurements. Readers are referred to Chotas *et al*.^3^ for additional details on the design, composition, and construction of the phantom and embedded test objects.

### B. Data acquisition

In order to assess the applicability of the phantom, digital radiographs were obtained at seven different techniques on five computed radiography systems and a direct radiography system (Table I).

**Table I:** Digital radiography systems and laser printers used for the evaluation of the QC phantom.

DR System	Manufacturer	Printer
FCR‐9501‐HQ	Fuji Medical Systems, Tokyo, Japan	Fuji FLIM‐D
AC3‐CS	Fuji Medical Systems, Tokyo, Japan	Imation Dryview 9800^a^
KESPR‐400	Eastman‐Kodak, Rochester NY	Kodak XLP
Thoravision	Philips Medical Systems, Best, The Netherlands	Polaroid Helios 1417^b^
ADC Compact	Agfa‐Gevaert, Morstel, Belgium	Agfa Matrix
CR 2000	Lumisys, Sunnyvale, CA	Philips EVL 1000 Laser Imager

a The Dryview 9800 is now supplied by Eastman‐Kodak, Rochester, NY.

b The Helios 1417 is now the Digital 400 supplied by Sterling Diagnostic Imaging, Greenville, SC, but is no longer available.

Images of the phantom were obtained at two different kVp settings, 81 and 117 kVp. Using one of the systems, the Fuji FCR‐9501‐HQ system, images were obtained using phototimed techniques at each kVp. Four additional images were then acquired at each kVp using approximately 1/5 and 5 times the phototimed mAs. For the other imaging systems, the radiographic techniques were adjusted to produce entrance skin exposures (ESE) similar to those measured for the Fuji FCR‐9501‐HQ system. Additional images were also obtained for each system using varying techniques currently utilized at our institutions. All images were obtained at 180 cm source to image distance (SID) using a conventional wall Bucky, except for the Philips Thoravision and the Fuji 9501, which are both integrated systems. The entrance exposure to the phantom was measured for each exposure using a Radcal 1515 exposure meter (Radcal Corp., Monrovia, CA) and a 6 cm^3^ ion chamber (Radcal 10×5–6) positioned in the midmediastinal region in front of the phantom. Because of this setup, backscattered radiation from the phantom was included in the exposure measurements. A linear gray‐scale transformation was applied to each image and any other post‐processing procedures applied by the systems were turned off. Films were produced of each image using the laser printer associated with the particular imaging system (Table I). Hard‐copy films were used to evaluate the spatial resolution and low‐contrast test objects because of the concern for the wide variations in the display quality of imaging workstations in soft‐copy presentations. The phantom manufacturer's protocol was used to evaluate the images, which included measurements of the average optical density, mean pixel value, and low‐contrast resolution in the heart, lung, and subdiaphragm regions, and spatial resolution in the lung region. For the average pixel value, the ROI function of the review workstations was used to obtain mean and standard deviations within the reference regions. The ROIs were placed within the center of the specified loop regions in the heart, lung, and subdiaphragm areas. The spatial resolution and the number of visible low‐contrast objects in each region were recorded by two independent observers. Spatial resolution was evaluated visually from the hard‐copy film using a 25×magnifier and a viewbox. The resolution test object was viewed under magnification and the smallest line pair object that could be resolved was recorded.

In addition to the phantom images, eight chest radiographs of actual patients, acquired with the Fuji 9501‐HQ system, were used to compare the phantom to actual patient images. All patient images were acquired at 115 kVp using phototimed techniques. For each patient image the mean pixel values and standard deviations were obtained from ROI's placed in similar locations as those in the phantom. Care was taken to use representative but relatively “clear” areas in the regions of the clinical images to minimize the dependence of the results on background anatomical variations.

### C. Relative signal evaluation

For each ROI in the lung, heart, and subdiaphragm regions of the phantom and patient images, signal levels were normalized relative to the lung region by calculating the ratio of plate exposure (derived from the average pixel values in the ROI) from each region $\left(E_{i}\right)$ to that in the lung region $\left(E_{L}\right)$. The pixel value to plate exposure relationship was provided by each manufacturer and in general had the form Q=a×log(b×E)+c, where *E* is the plate exposure, *Q* is the pixel value, and *a, b, c* are system‐specific constants. The normalized or relative signal levels were calculated using the equation

(1) $$S_{i}=\frac{E_{i}}{E_{L}}=10^{(Q_{i}-Q_{L})/M},$$

where $Q_{i}$ is the mean pixel value from region *i*, $Q_{L}$ is the mean pixel value from the lung region, and *M* is a system dependent proportionality factor. For the Fuji systems, *M* was set equal to 1024/*L*, where *L* is the latitude of the image reported by the system. For the Agfa systems, M=1157.51, determined empirically. For the Kodak^4^ and Lumisys^6^ systems, *M* was set to 1000. Since digital images were not available for the Fuji 9501 and Philips Thoravision, a relative signal could not be computed so these units were omitted from the relative signal evaluation.

### D. Histogram analysis

To compare the phantom images to the patient images, the area normalized signal or pixel value frequency histograms showing the relative exposures at the plate were generated for images acquired with the Fuji system. Pixel values in each image were converted to relative exposure values using

(2) $$\log\left(\frac{E}{c}\right)=\frac{L}{1024}(511-Q)-\log(S)$$

where *L* is the latitude of the image, *Q* is the image pixel value, *S* is the Fuji exposure indicator or sensitivity value, and *c* is a constant. Histograms for the patient images were filtered with a low‐pass filter to remove high frequency sampling noise present in the data. The sampling noise is introduced by the Fuji CR reader during a two‐stage, down‐sampling processing step where the image data is converted from 12‐bit to 11‐bit and subsequently to 10‐bit pixel representation. The shapes of the patient histograms were compared to the phantom histogram and the idealized chest histogram used by the Fuji CR processing software.^5^

## III. RESULTS AND DISCUSSION

### A. Resolution and contrast detail sensitivity

Table II lists the measured spatial resolution and entrance skin exposures from each imaging system. The spatial resolution measurement for all digital radiography systems was lower than but related to the Nyquist frequency of the systems. The pixel size in most current digital radiography systems is between 100 to 200 *μ*m,^1^ depending on cassette size. For a 35×43 cm cassette, the pixel size for the Kodak, Agfa, and Lumisys readers was 0.17 mm/pixel, and 0.2 mm/pixel for the Fuji and Philips readers. For these pixel sizes, the frequency range and increments of line pair patterns of the phantom are sufficient to reveal degradation in the resolution response of the system. Spatial resolution was highest for the Fuji 9501‐HQ system at 4 lp/mm. For the Agfa and Kodak units, spatial resolution was generally around 2.8–3.0 lp/mm depending on technique. At 81 kV, 1.1 *μ*C/kg (4.3 mR) ESE, the image from the Kodak system was extremely noisy and showed large areas of pixel dropout due to insufficient exposure, particularly in the subdiaphragm area. The Philips Thoravision showed spatial resolution ranging from 2.6–2.7 lp/mm. At the low exposure technique obtained at 81 kVp, the image was nonuniform and mottled due to insufficient exposure, and the minimum line pair in the test pattern was unresolved. Artifacts introduced by inappropriate image processing at very low exposures obscured the visibility of the test objects for the Thoravision and KESPR‐400 systems. In general, images acquired at the low technique showed slightly lower resolution due to quantum mottle noise for all systems.

**Table II:** Measured spatial resolution, entrance skin exposures (ESE) and low contrast resolution evaluation for the lung, heart, and subdiaphragm regions. Numbers for the low‐contrast resolution indicate the number of disks visible in each of the heart, lung, and subdiaphragm regions. Columns shown in bold are the clinically used techniques for each system. For entries marked “NA” the test object was not visible in the image.

Fuji AC3‐CS							
kVp	117	117	**117**	81	81	81	
ESE (mR)	5	**29.5**	192.8	3.8	21.5	111	
LP/mm	2.4	3	3	2.3	3	3	
Lung	6	22	**22**	6	12	12	
Heart	6	**10**	11	6	12	14	
Diaphragm	8	**15**	17	5	1319		
Fuji FCR 9501‐HQ							
kVp	117	117	117	81	81	81	**115**
ESE (mR)	5.1	28.4	175.5	3.6	23.5	113.7	**67**.
LP/mm	3	4	4	2.3	4	4	**4**
Lung	11	11	14	8	13	16	**12**
Heart	6	14	15	8	13	18	**15**
Diaphragm	10	18	21	NA	15	21	**17**
Agfa Compact							
kVp	117	117	117	81	81	81	^120^
ESE (mR)	8.08	29	160	3.74	21.1	102.5	**25.1**
LP/mm	2.8	2.8	3	2.6	2.8	2.8	**2.8**
Lung	9	7	7	6	7	8	7
Heart	8	11	14	8	12	15	**11**
Diaphragm	13	17	21	5	14	19	**14**
Kodak KESPR‐400							
kVp	117	**117**	117	81	81	81	
ESE (mR)	5.8	**26.6**	173.7	4.3	22.6	113.7	
LP/mm	2.5	**2.8**	3	NA	2.8	3	
Lung	11	**12**	13	NA	12	13	
Heart	11	**15**	18	6	10	19	
Diaphragm	15	**19**	22	NA	15	22	
Philips Thoravision							
kVp	117	117	117	81	81	81	**150**
ESE (mR)	5.3	27.2	182.3	3.8	21.6	102.3	**39.5**
LP/mm	2.3	2.6	2.7	NA	2.6	2.7	**2.7**
Lung	9	14	11	6	13	14	**11**
Heart	9	12	17	6	13	17	**12**
Diaphragm	13	20	22	6	17	22	**20**
Lumisys CR 2000							
kVp	117	117	117	81	81	81	**120**
ESE (mR)	5.2	27.4	174.8	3.74	22	107.4	**53.4**
LP/mm	2.5	2.5	2.5	2.4	2.5	2.5	**2.6**
Lung	8	6	7	4	8	7	**8**
Heart	8	11	10	5	10	12	**12**
Diaphragm	12	15	18	7	12	16	**16**

**Figure 3:**
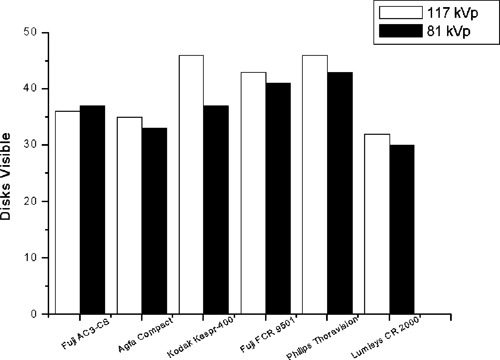
Number of disks visible (lung, heart, and subdiaphragm regions) for the phototimed technique for each system.

Figure 3 illustrates the sum of disks visible in the lung, heart, and subdiaphragm regions for just the phototimed techniques. There were 25 disks present in each of the three phantom test objects (75 total disks present).

The phantom shows somewhat similar low‐contrast performance across each of the systems, with the Kodak, Fuji 9501, and Philips systems resolving slightly more disks. The variation may be a result of window/level settings and differences in the output of the laser printers used, which was not controlled. The phantom appears to be useful for tracking low contrast visibility for a variety of systems, as it does not exhibit overwhelmingly better or worse performance on any particular system, and targets an appropriate range of visibility for radiographic applications. Results from the low‐contrast evaluation are listed in Table II. The number listed for each technique gives the number of disks visible in each of the lung, heart, and subdiaphragm test objects.

For the lung and heart fields, typically 3–4 disks were seen in the first three rows and 0–2 disks visible in the two smallest rows (1.0 and 0.5 mm diameter). This suggests that the contrast range of the disks is suitable for spotting changes in contrast detail sensitivity. For the subdiaphragmatic area, there were typically 4–5 disks visible in each row except for the smallest row (0.5 mm diameter). Therefore, the contrast ranges for the diaphragm region may not be sensitive enough to detect changes in contrast detail sensitivity for under‐exposed regions. Contrast detail sensitivity improved with higher exposures, and the reverse was true for lower exposures (low mAs techniques).

### B. Relative signal evaluation and histogram analysis

**Figure 4:**
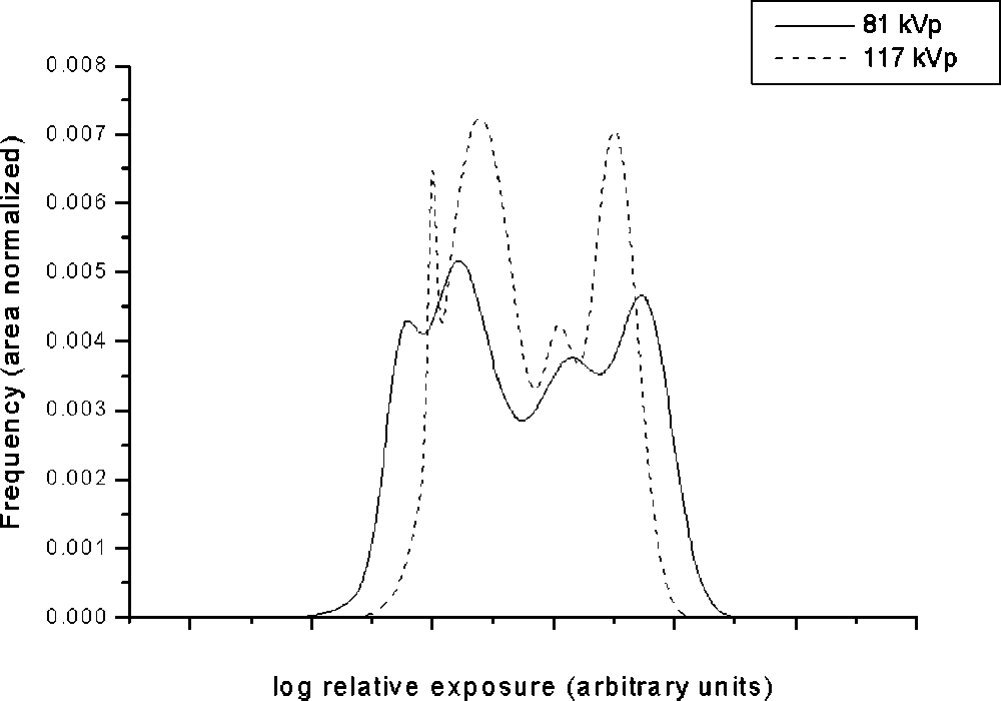
Phantom histograms at 81 and 117 kVp. The histograms were normalized to have equivalent areas. The locations of each histogram were adjusted to better illustrate the change in dynamic range from 81 to 117 kVp.

The relative signal values for phantom images are tabulated in Table III. The subdiaphragm region of the 81 kVp low exposure technique obtained on the Kodak system was left out of the analysis because the test pattern was unresolved due to the extremely low exposure in this region. The ratio of signal levels in the heart and subdiaphragm regions to the lung tended to be slightly higher at 117 kVp relative to those at 81 kVp. This was expected based on decreased subject contrast at higher beam energies. As the *x*‐ray beam energy increased, the difference in signal level decreases and the histogram became compressed due to decreasing subject contrast (see Fig. 4). This resulted in an increase in the relative signal levels at higher beam energies. The general similarity of the signal level ratios with imaging system indicates that the phantom can produce consistent results for different systems. This is a positive attribute for implementing QC programs at institutions with a heterogeneous mix of digital radiographic systems. Table IV shows the relative signal values for the patient images.

**Table III:** Relative signal for the chest phantom ±1 SD averaged over the low, middle, and high exposure images. Signal levels were normalized to that in the lung. The average relative signal for the Kodak KESPR‐400 unit at 81 kVp excludes the low exposure image due to extremely low signal level.

	Fuji AC3‐CS		Agfa Compact		Kodak KESPR‐400		Lumisys CR 2000	
kVp	Heart	Diaphragm	Heart	Diaphragm	Heart	Diaphrag	Heart	Diaphragm
117	0.70±0.01	0.33±0.01	0.65±0.01	0.24±0.01	0.65±0.02	0.27±0.02	0.69±0.03	0.26±0.01
81	0.61±0.02	0.26±0.04	0.05±0.01	0.13±0.03	0.52±0.01	0.16±0	0.56±0.01	0.16±0.05

**Table IV:** Relative signal levels for patient images. Signal levels were normalized to that in the lung.

	p1	p2	p3	p4	p5	p6	p7	p8	Average
kVp	115	115	115	115	115	115	115	115	115
Heart	0.19	0.32	0.22	0.14	0.12	0.25	0.20	0.23	0.21±0.06
Diaphragm	0.06	0.16	0.10	0.06	0.13	0.11	0.09	0.09	010±0.03

**Figure 5:**
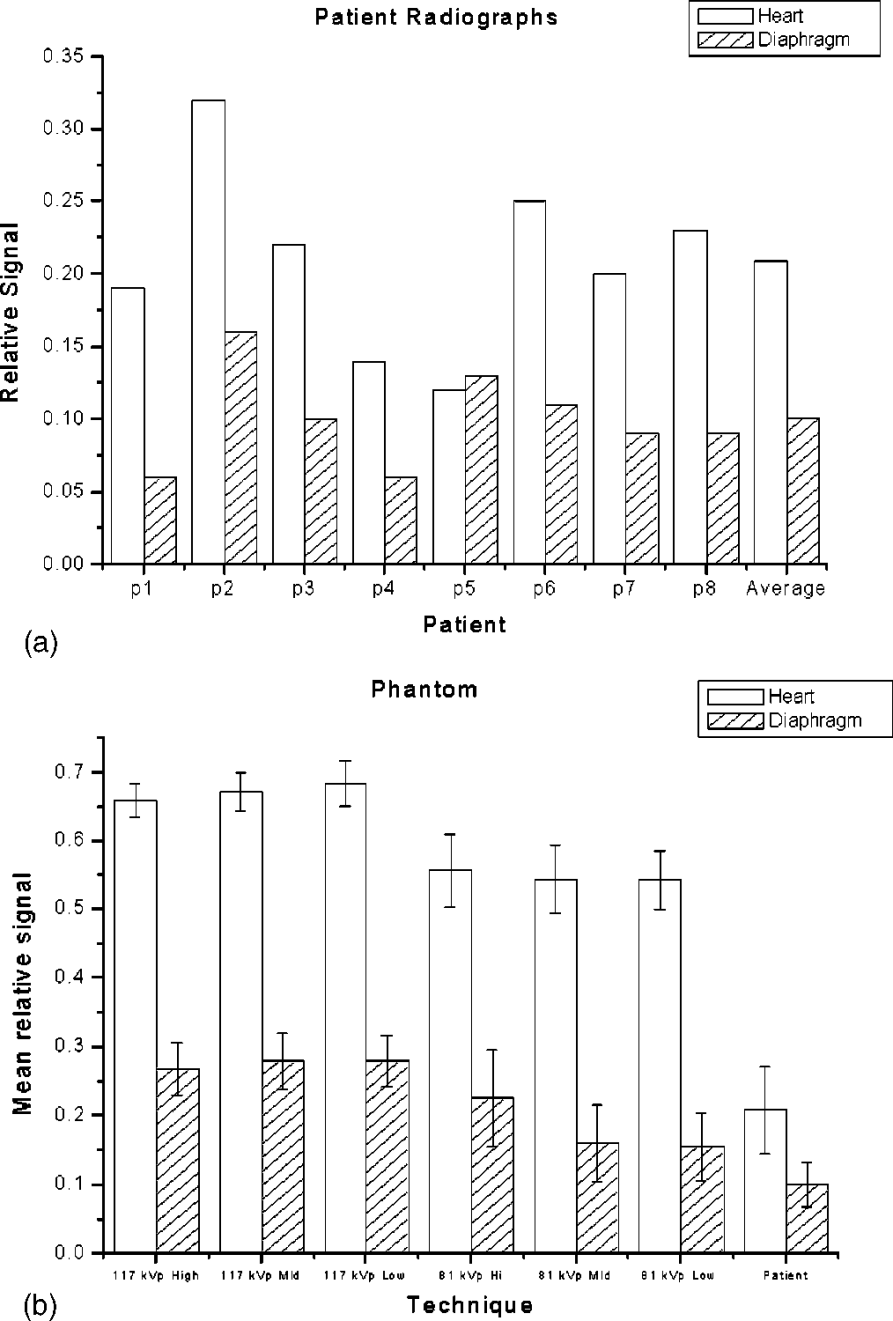
(a) Average relative signal levels for the patient radiographs. (b) Average relative signal levels for the phantom radiographs.

Figure 5 shows bar graphs of the average relative signal level for the patient chest radiographs [5(a)] and the imaging systems [5(b)]. In phantom images, the ratio of the heart to sub‐diaphragm relative signal level was 2.43±0.14, very similar to that in patient images (2.07±0.44). Thus the relative attenuation between the heart and subdiaphragm regions of the phantom is a good match to the attenuation differences found in actual patients. However, the absolute value of the average lung‐relative signal level for the heart region was 3.22±0.31 times higher in phantom images (0.67±0.03) compared to that in patient images (0.21±0.06). For the sub‐diaphragm region, the average relative signal in the phantom images was 2.75±0.34 times higher than that in patient images (0.28±0.04 versus 0.10±0.03, respectively). The results suggest that these regions of the phantom are not attenuating enough with respect to the lung to generate histograms more similar to those of real chest radiographs.

Histograms of two phantom images using phototimed techniques at 81 and 117 kVp acquired with the Fuji AC3 system are shown in Fig. 4. Area normalized histograms of the relative log‐exposure to the plate for the phantom images show four distinct peaks corresponding to the different regions of the phantom (lung, heart, subdiaphragm, and unattenuated region).

**Figure 6:**
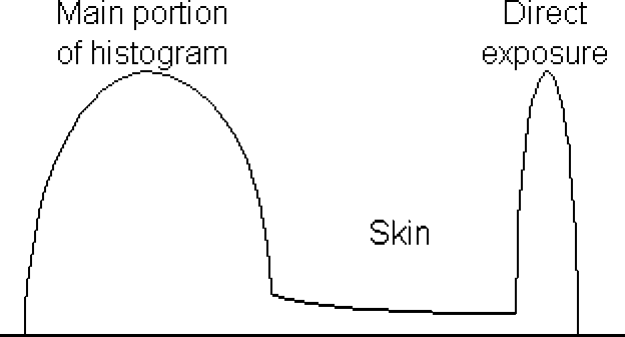
Generalized Fuji histogram illustrating the histogram segments corresponding to different image areas.^5^ The graph is meant to show the parts of the histogram which correspond to various anatomical regions and do not necessarily reflect the actual histograms used by the Fuji image processing software.

The generalized chest histogram used by Fuji^5^ (Fig. 6) consists of two regions, a broad peak of the main image data and a sharp narrow peak representing the directly exposed areas of the image. A valley representing the skin and other low attenuation regions separates the two peaks. No valley that would correspond to skin or soft tissue is present in the histogram of the phantom images. This deficiency, however, did not appear to affect the quality of post‐processed images. The effect of kVp on the phantom histograms is apparent. Shifting to higher kVp decreases the dynamic range of the image, making the histogram narrower. The histogram is also shifted to the right, towards higher exposure values.

**Figure 7:**
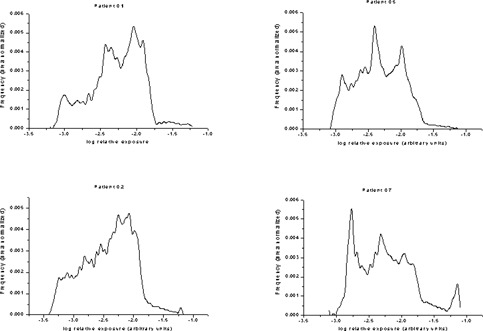
Sample of the histograms from patient radiographs.

Examples of area‐normalized histograms of the patient images are illustrated in Fig. 7. These histograms showed wide variations in shape and range, depending on the size, physical condition and image quality of the final image. Broad peaks representing the different fields are seen in some of the patient histograms. Also visible is the valley corresponding to the skin and other low attenuating tissues. The breadth of the histograms of the phantom and the patient images suggests that they have significantly different dynamic ranges. The scalar value of the dynamic range or latitude of Fuji CR images is reported by the system in the *L* parameter associated with the image, where *L* is the $\log_{10}$ of the dynamic range. For the patient images at 115 kVp, the average *L* value was 2.38±0.20 while for the phantom images at almost the same kVp (i.e., 117), the average L value was 1.6±0.0.

Although the phantom was designed to resemble a chest radiographically, the phantom does not look exactly like a chest. The ribs are composed of linear structures with sharp, well‐defined edges, and the mesh pattern is visible across the phantom. However, the presence of these linear structures has shown to be of some benefit. During the evaluation period, a subtle blurring artifact appearing as a slightly darkened band was observed on clinical images coming from an Agfa CR reader. The appearance of the artifact was often masked by normal anatomical variations in the image, but could still be observed with window/level changes. Using the phantom to evaluate the system, it was discovered that the artifact was due to a pixel shift, clearly visible in the sharp mesh lines of the phantom images. This incident demonstrated the usefulness of the phantom in identifying and characterizing subtle artifacts that might otherwise be masked by variations in patient anatomy.

## IV. CONCLUSION

Just as film processors require a routine quality control program to monitor processing quality for film, digital radiography systems also require a quality control program to monitor image quality (e.g., changing exposure conditions and hardware deterioration). In this study, a QC phantom designed for this purpose was evaluated. The phantom was evaluated using six digital radiography systems from five different manufacturers. Phantom characteristics were similar across each of the imaging systems with respect to relative signal levels in different areas of the phantom images. The line pair test object provided a sufficient range of line pairs to evaluate and monitor the Nyquist frequency of the DR systems used in the study. The line pair test object is not expected to be able to detect small or subtle changes in the optical system of CR systems. Such changes would be more appropriately detected by measuring the MTF of the CR system, which would be beyond the scope of a routine QC program. With two of the DR systems, the image processing appeared to be unable to deal with extremely low exposures and high quantum mottle in the images, producing artifacts that obscured the visibility of the test objects. There was variation in the low‐contrast performance of the phantom across the DR systems used, but the low contrast characteristics of the phantom were felt to be reasonably similar for all systems. Use of standardized window/level settings and exposure technique may help to reduce variability in the results. While the low contrast objects were able to detect contrast changes over a wide range of exposures, the sensitivity of the low contrast objects to exposure was not investigated. Both the spatial resolution object and background mesh should help identify problems with laser and components involved in the scanning process. Attenuation properties of the phantom were found to be somewhat different from actual chest radiographs. The phantom produces images with a much narrower dynamic range than is found with clinical chest images. However, these differences did not appear to be problematic in the phantom's intended use. Further work is required to track the long‐term capabilities of the phantom and the sensitivity of the phantom to detect changes in the DR system. Overall, the results suggest that the phantom can be an effective tool for a routine QC program for diagnosing image artifacts and monitoring the performance and image quality of digital radiography systems.

## ACKNOWLEDGMENTS

The authors would like to thank Nuclear Associates for providing the chest phantom evaluated in this study.

## References

[1] J. A. Seibert et al., Acceptance Testing and Quality Control of Photostimulable Storage Phosphor Imaging Systems, Draft document, American Association of Physicists in Medicine Task Group #10.

[2] M. Freedman , D. Steller , H. Jafroudi , and S. K. Mun , “Quality Control of Storage Phosphor Digital Radiography Systems,” J. Digit Imaging 8, 67–74 (1995). 10.1007/BF03168129 7612704

[3] H. G. Chotas , C. E. Floyd , G. A. Johnson , and C. E. Ravin , “Quality Control Phantom for Digital Chest Radiography,” Radiology 202, 11–116 (1997). 10.1148/radiology.202.1.8988199 8988199

[4] M. Bogucki , D. P. Trauernicht , and T. E. Kocher , “Characteristics of a Storage Phosphor System for Medical Imaging,” Kodak Health Sciences Division Technical and Scientific Monograph No. 6.

[5] N. Nakajima , H. Takeo , M. Ishida , and T. Nagata , “Automatic Setting Functions for Image Density and Range in the FCR System,” Fuji Computed Radiography Technical Review No. 3.

[6] T. M. Bogucki , private communication.

